# Transcriptome visualization and data availability at the *Saccharomyces* Genome Database

**DOI:** 10.1093/nar/gkz892

**Published:** 2019-10-15

**Authors:** Patrick C Ng, Edith D Wong, Kevin A MacPherson, Suzi Aleksander, Joanna Argasinska, Barbara Dunn, Robert S Nash, Marek S Skrzypek, Felix Gondwe, Sagar Jha, Kalpana Karra, Shuai Weng, Stuart Miyasato, Matt Simison, Stacia R Engel, J Michael Cherry

**Affiliations:** 1 Department of Genetics, Stanford University, Palo Alto, CA 94304-5477, USA; 2 Oregon Health Sciences University, Portland, OR 97239, USA

## Abstract

The *Saccharomyces* Genome Database (SGD; www.yeastgenome.org) maintains the official annotation of all genes in the *Saccharomyces cerevisiae* reference genome and aims to elucidate the function of these genes and their products by integrating manually curated experimental data. Technological advances have allowed researchers to profile RNA expression and identify transcripts at high resolution. These data can be configured in web-based genome browser applications for display to the general public. Accordingly, SGD has incorporated published transcript isoform data in our instance of JBrowse, a genome visualization platform. This resource will help clarify *S. cerevisiae* biological processes by furthering studies of transcriptional regulation, untranslated regions, genome engineering, and expression quantification in *S. cerevisiae*.

## INTRODUCTION

The annotation of >6000 genes in the reference genome of *Saccharomyces cerevisiae* is maintained by the *Saccharomyces* Genome Database (SGD; www.yeastgenome.org) (1), and is based on the common laboratory strain S288C (2). As a model organism database, SGD maintains a record of the sequence and chromosomal location of these gene features and manually curates functional annotation of their protein products in accordance with the guidelines of the Gene Ontology consortium (GO; www.geneontology.org) (3). This sequence information can be used to determine homology relationships across other organisms, and GO provides a controlled vocabulary and relational ontology for describing molecular functions, biological processes, or cellular components that may be shared by evolutionary conservation.

Precise gene mRNA sequence and coordinates are relevant to studies of mRNA stability (4), localization (5), and translational efficiency (6). Additionally, genome engineering projects seeking to alter or to vectorize the expression of *S. cerevisiae* genes (7) and transcript-based computational methods for measuring gene expression could also benefit from categorization of full-length mRNA transcripts (8). High-throughput next generation sequencing methodologies that measure the RNA expression of genes or map protein regulation of genomic DNA have become increasingly sensitive, making identification of these sequences easier.

In this paper we describe how SGD has taken data files and associated metadata from these RNA sequencing (RNA-seq) experiments, available at public repositories such as the Gene Expression Omnibus (GEO; www.ncbi.nlm.nih.gov/geo/) (9) and Array Express (www.ebi.ac.uk/arrayexpress/) (10), and visualized them using JBrowse (jbrowse.org) (11), a web-based genome browser application. We have divided datasets into tracks that either map assay values continuously across each position of the genome or highlight regions of interest identified experimentally. One of the categories of biochemical assays represented in SGD’s JBrowse instance is the transcriptome: the identification of all RNA transcripts produced from the entire genome under particular conditions. The 5′ and 3′ untranslated regions flanking each gene are captured in these data tracks, where we aim to provide the research community with additional information about fundamental yeast cellular transcription.

## INTEGRATION OF TRANSCRIPTOME DATA

A number of publications have separately sequenced the 5′ and/or 3′ ends of transcripts in *S. cerevisiae* (12–21). SGD has provided data from these studies to map the 5′ and 3′ boundaries of mRNAs. However, Pelechano *et al.* developed Transcript Isoform Sequencing (TIF-seq) to characterize each individual transcript of the S288C strain (22). With this method, both ends of a single RNA molecule are identified at the same time. Thus, single complete transcripts are determined, rather than being inferred by matching the 5′ and 3′ ends that have been sequenced from different experiments. We have incorporated the data from Pelechano's study of two separate metabolic conditions (glucose- or galactose-containing media) to generate a representative view of the *S. cerevisiae* transcriptome (23).

First, we downloaded a text file of transcript coordinates and raw counts from GEO (accession GSE39128). We selected the subset in which each transcript's chromosomal location fully overlapped the protein coding sequence of a single open reading frame (ORF) annotated by SGD on the same strand. In order to compare across conditions, we combined transcripts from both conditions and gave them unique identifiers containing the systematic name of the associated SGD ORF. We first ordered transcripts by distance upstream of the start site of the associated ORF and then in descending order by transcript length, and finally created an output file using the General Feature Format (GFF) annotation format. Transcript identifiers are consistent across all files. For example, the YAL008W_id199 transcript isoform in the file with the most abundant transcripts found in yeast grown in glucose media (most_abundant_full-ORF_transcripts_ypd.gff3) corresponds to the same isoform in all other files; most_abundant_full-ORF_transcripts_gal.gff3, which contains the most abundant transcripts found under the galactose condition.

To begin to define a transcriptome, we initially created a set with all full-length transcripts for all ORFs (unfiltered_full-ORF_transcripts.gff3). Because growth condition affects what is being transcribed, we split this set into two different transcript sets, based on growth conditions (galactose or glucose). To depict the full range of what is transcribed for a particular ORF, for each growth condition, we then filtered the dataset for the longest transcript for each ORF. Finally, to indicate the predominant transcript isoform under each condition, we also created a most abundant transcript set. The GFF annotation filename suffixes for the longest and most abundant transcript sets denote whether they refer to the glucose nutritional condition (_ypd.gff3) or galactose nutritional condition (_gal.gff3). All filenames and descriptions can be found in Table 1.

**Table 1.** Filenames and descriptions of data tracks for transcript isoforms (GFF3 format) and coverage (bigWig format)

Data Track Filename	Description
longest_full-ORF_transcripts_ypd.gff3	This track contains the longest transcript overlapping each individual ORF completely for WT cells grown in glucose (ypd) media.
longest_full-ORF_transcripts_gal.gff3	This track contains the longest transcript overlapping each individual ORF completely for WT cells grown in galactose (gal) media.
most_abundant_full-ORF_transcripts_ypd.gff	This track contains the most abundant transcript overlapping each individual ORF completely for WT cells grown in glucose (ypd) media.
most_abundant_full-ORF_transcripts_gal.gff	This track contains the most abundant transcript overlapping each individual ORF completely for WT cells grown in galactose (gal) media.
unfiltered_full-ORF_transcripts.gff3	This track contains all transcripts that overlapped individual open reading frame (ORF) completely for WT cells grown in either glucose (ypd) or galactose (gal) media.
plus_strand_coverage_ypd.bw	For WT cells grown in glucose media (ypd), the amount of transcripts covering each position on the plus strand is represented in this track.
plus_strand_coverage_gal.bw	For WT cells grown in galactose media (gal), the amount of transcripts covering each position on the plus strand is represented in this track.
minus_strand_coverage_ypd.bw	For WT cells grown in glucose media (ypd), the amount of transcripts covering each position on the minus strand is represented in this track.
minus_strand_coverage_gal.bw	For WT cells grown in galactose media (gal), the amount of transcripts covering each position on the minus strand is represented in this track.

To reflect how many transcripts covered each individual nucleotide of the *S. cerevisiae* genome, coverage tracks were generated for each condition from the raw transcript text file. Because of the size of the files, we split transcript coverage into plus and minus strands and created separate bigWig (.bw) files. The presence of intergenic, truncated and polycistronic transcription are also reflected in the provided files (Table 1).

## TRACK VISUALIZATION AND ANNOTATION DOWNLOAD

SGD’s JBrowse instance is accessible through the ‘Genome Browser’ link within the ‘Sequence’ menu in the purple toolbar that runs across the top of most SGD webpages, or via direct URL (browse.yeastgenome.org). Within the JBrowse browser window, the TIF-seq transcriptome tracks can be viewed by using the ‘Select tracks’ button in the top left corner. In the resulting slide out window, nine data tracks comprise the transcriptome (unfiltered transcripts that fully overlap ORFs, longest transcript in each of two conditions, most abundant transcript in each of two conditions, and both plus and minus strand coverage in each of two conditions), and can be navigated to in several ways using the categorical track selector to the left or the text query box at the top. Choosing ‘Pelechano’ within the ‘First Author’ category or ‘23615609’ within the ‘PMID’ category and checking the leftmost boxes for each track in the metadata display table results in the tracks being viewable in the JBrowse navigation window. These instructions are also reviewed in a video tutorial on SGD’s YouTube page (www.youtube.com/SaccharomycesGenomeDatabase). A recent post on the SGD Blog (www.yeastgenome.org/blog/explore-the-s288c-transcriptome-in-jbrowse) has the YouTube tutorial embedded and provides direct links to the tracks in JBrowse, and includes a direct download link to a zipped folder of all the track files (https://sgd-dev-upload.s3.amazonaws.com/S000246061/Pelechano_2013_PMID_23615609.zip).

**Figure 1.**
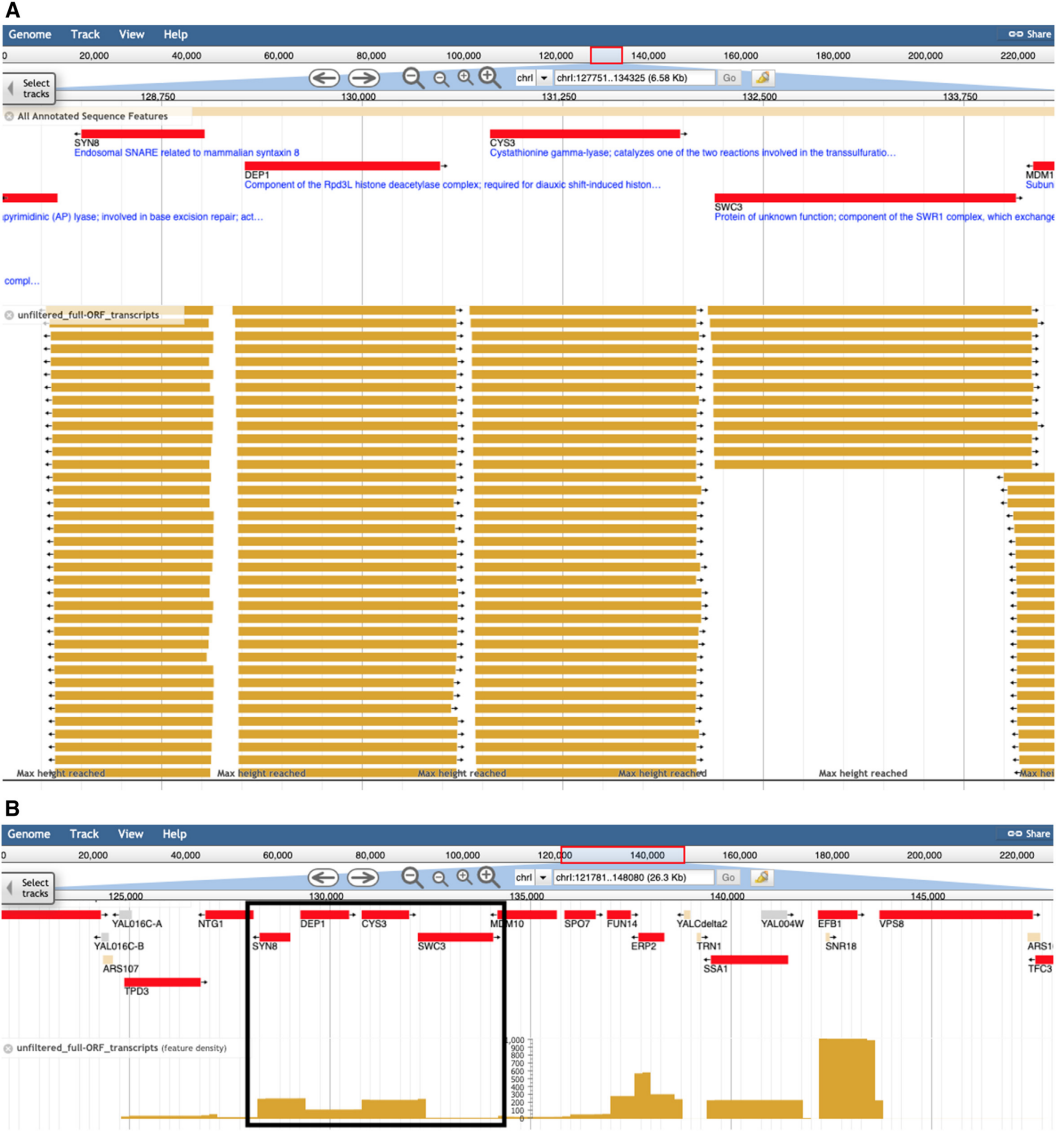
Unfiltered transcript isoforms that overlap ORFs displayed at various levels of zoom in JBrowse. (**A**) Individual glyphs representing each transcript isoform are visible at close zoom. (**B**) At lower magnification, the same region (outlined in the box) is displayed as a collapsed histogram.

**Figure 2.**
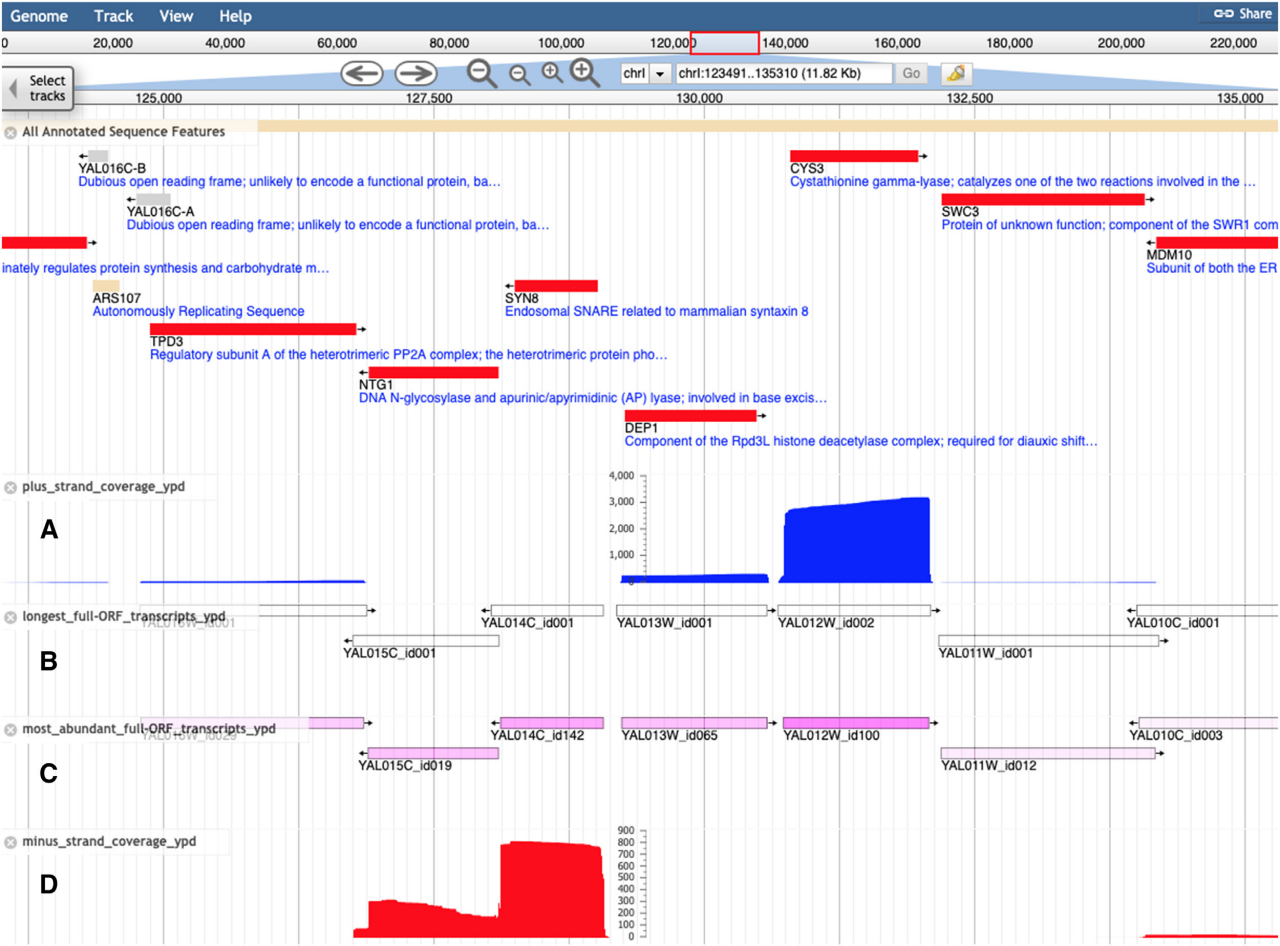
Representative tracks for transcript isoforms and coverage in glucose containing media. (**A**) Plus-strand transcript coverage in blue. (**B**) Longest transcript isoform for each ORF. (**C**) Most abundant transcript isoform for each ORF. (**D**) Minus-strand transcript coverage in red.

**Figure 3.**
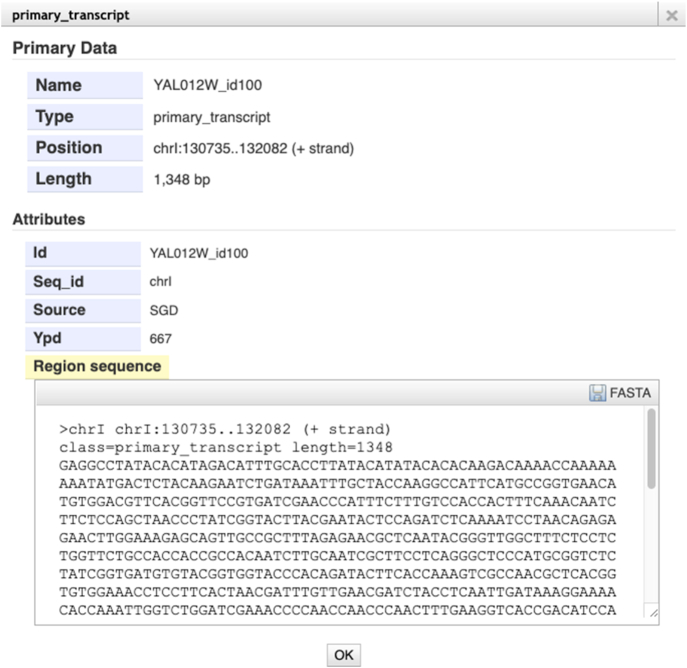
Transcript isoform dialog popup. The ‘Ypd’ or ‘Gal’ attribute lists the raw abundance in the glucose- or galactose-media condition, respectively. ‘Region sequence’ displays the predicted sequence based on the S288C reference genome.

Once displayed in the JBrowse navigation window, tracks can be distinguished by color. Unfiltered transcripts are displayed in solid yellow and can number up to the hundreds. By default, the browser clips the number of transcripts viewed at close zoom or collapses the track into a ‘density’ view at far zoom (Figure 1). For the most abundant and longest transcript tracks, transcript identifiers are displayed beneath the glyph (Figure 2B, C). Pink shading represents the logarithmically scaled transcript abundance; darker shading reflects higher abundance. Clicking on the glyph for a transcript reveals a popup listing its exact coordinates, raw abundance in the particular media condition, and predicted sequence based on the reference genome (Figure 3). Quantitative coverage tracks for each condition are presented as histograms; blue for the plus strand and red for the minus strand (Figure 2A, D). These tracks represent the cumulative raw abundances of all transcripts at each position of the S288C reference sequence.

## FUTURE DIRECTIONS

There are multiple ways to expand the transcriptome data that SGD provides. Pelechano's dataset examines transcripts and their abundance at specific glucose/galactose concentrations for mid-log phase cells. However, additional datasets, such as those from experiments that examine conditions utilizing different chemical, genetic or epigenetic perturbations, as well as extended time courses, can be incorporated. Large heterogeneity between individual transcripts for the same gene was a key observation of the Pelechano study. Incorporation of single cell sequencing methodologies could clarify the varied transcriptional landscape between individual cells and determine the existence of burgeoning subpopulations over time (24,25). Multiple studies exist that also profile the transcriptional heterogeneity of untranslated regions (UTR) and transcription start sites (TSS) utilizing alternative deep sequencing technologies (26,27). Overlaying existing ribosome profiling (Ribo-seq) studies with the transcriptome data could expand our understanding of transcriptional dynamics (28). SGD will continue to integrate the aforementioned research in a systematic way and depict them informatively to help to gain insight into the *S. cerevisiae* transcriptome.

## DATA AVAILABILITY

JBrowse is an open source genome browser available in the GitHub repository (https://github.com/GMOD/jbrowse). SGD software is open source and available from the GitHub repository (https://github.com/yeastgenome). The TIF-seq data from Pelechano *et al.*, 2013, is accessible at NCBI GEO archive (accession GSE39128).

## Notes

Database URL: www.yeastgenome.org
